# Global retrospective analysis of clinician- and patient-reported clinical characteristics and humanistic burden of patients with gastroesophageal cancers on first-line treatment

**DOI:** 10.1186/s12885-023-10553-7

**Published:** 2023-02-23

**Authors:** Hong Xiao, David Bertwistle, Keerun Khela, Chloe Middleton-Dalby, Jennifer Hall

**Affiliations:** 1 Bristol Myers Squibb, 3401 Princeton Pike, D.3243C, Lawrence Township, NJ 08648 USA; 2 Bristol Myers Squibb, Uxbridge, UK; 3 Adelphi Real World, Bollington, UK

**Keywords:** Gastric cancer, Esophageal adenocarcinoma, Gastroesophageal junction cancer, Quality of life, Disease burden, Retrospective, Multinational, Multicenter, Chart review (9/10 allowed)

## Abstract

**Background:**

Gastric cancer (GC), gastroesophageal junction cancer (GEJC), and esophageal adenocarcinoma (EAC), together, are leading causes of cancer deaths worldwide. Patient health-related quality of life (HRQoL) and well-being has become increasingly important alongside traditional oncologic outcomes for both patients and clinicians and may aid treatment decisions. We conducted a survey to examine the clinical characteristics, humanistic burden, and the effects of first-line (1L) treatment in patients with GC/GEJC/EAC, across different geographic regions, to address the paucity of real-world data.

**Methods:**

Clinicians treating patients with unresectable advanced or metastatic GC/GEJC/EAC in China, France, Germany, Japan, the United Kingdom, and the United States, during April–October 2019, were invited to provide data on their patients’ demographics, clinical characteristics, treatment, and HRQoL via medical chart reviews, clinician surveys, and patient questionnaires. Data were analyzed using descriptive statistics, regression analyses comparing active treatment and best supportive care. Patients were also stratified into subgroups that were identified either as human epidermal growth factor receptor 2 (HER2) positive, HER2 negative (which has a higher prevalence but for whom there are limited treatment options), or unknown HER2 status.

**Results:**

Survey data were analyzed for 995 patients, 87% of whom were on active treatment, most commonly dual or triple chemotherapy. Demographics and clinical characteristics were similar across countries with most patients having GC and the lowest incidence of GEJC and EAC in China. Overall, most patients had de novo disease with good response to 1L treatment, while their HRQoL and well-being was significantly worse than the general population. In 682 patients on active treatment with HER2 negative or unknown status, HRQoL also appeared to be worse in those with recurrent disease. Regression analysis identified several drivers of treatment decisions and factors impacting patients’ HRQoL, including stage of disease and comorbidities.

**Conclusions:**

In patients with advanced GC/GEJC/EAC, screening and assessment of HER2 status as well as patient-reported HRQoL outcomes are invaluable in aiding treatment decisions. The introduction of appropriate therapy soon after diagnosis has the prospect of achieving improved HRQoL and survival in these patients.

## Background

The demarcation between gastric and esophageal adenocarcinomas (EAC), and the classification of adenocarcinomas spanning the gastroesophageal junction, remain unclear [1]. Cases of gastric cancer (GC) and gastroesophageal junction cancer (GEJC) are frequently combined in incidence statistics: together, they represented the fifth most frequently diagnosed cancers (5.6% of all cancers) in 2020, with over a million new cases globally, and the fourth leading cause of cancer deaths, with 770,000 estimated deaths (7.7%) worldwide [2]. Esophageal cancer includes two distinct histologic subtypes, EAC and esophageal squamous cell carcinoma (ESCC), which have quite different etiologies and anatomic distributions with EAC occurring predominantly in the lower esophagus near the gastric junction, while ESCCs are mainly found in the upper and mid-esophagus. Together, EAC and ESCC accounted for over 600,000 cases (3.1% of all cancers) and over 500,000 deaths (5.5% of all deaths) in 2020 [2] with incidence rates of EAC rising sharply in developed countries over the past few decades [3]. In view of their molecular similarities, GC, GEJC and EAC are frequently grouped together as gastroesophageal adenocarcinomas [1].

Early stages of GC/GEJC/EAC typically present with minimal or no symptoms; for this reason, these cancers are frequently diagnosed at an advanced stage, resulting in poor prognosis [4], with a 5-year overall survival rate in the US (considering all stages) of 20–30% [5]. A higher survival rate in Japan (52%) is attributed to early screening programs [6]. However, the incidence varies dramatically, with more than two-thirds of all cases occurring in developing countries, and reports of up to 43% of cases occurring in China [6]. While lacking in most countries, particularly those with a low incidence of GC/GEJC/EAC, mass screening programs in the Asian countries, Korea and Japan, have been shown to be cost-effective and widely available, leading to more frequent early detection [7].

Although 5-year survival remains a critical effectiveness measure in the medical care of patients with GC/GEJC/EAC, the consideration of patient health-related quality of life (HRQoL) data is becoming paramount when determining the appropriate treatment strategy [8, 9]. HRQoL is a multidimensional construct with functional domains that can be described by three categories: physiological, psychological, and social. In a patient with GC/GEJC/EAC, a physiological effect might be nausea or problems with swallowing, a psychological effect could be depression, and a social effect might be withdrawal due to embarrassment about being ill [10].

Patients with advanced GC/GEJC/EAC are generally offered palliative systemic therapy to prolong survival and provide symptom relief, but also to improve or maintain health-related HRQoL [11]. Several studies have shown that baseline HRQoL can predict survival outcomes in patients with advanced GC/GEJC/EAC in both 1L and second-line (2L) settings. These studies have found that better physical function and ability to work or perform daily activities, as well as overall HRQoL, are associated with significantly better prognosis [12]. Indeed, HRQoL in patients with GC has been increasingly included as an outcome measure in clinical research, usually to evaluate the effects of medical treatment [10]. Hence, although there are numerous reports of HRQoL assessments in controlled trials in advanced GC [11], the published literature describing the burden of disease in patients in a real-world setting and across different healthcare systems is scant.

The aim of this multinational survey was to aid an understanding of the impact of advanced GC/GEJC/EAC and its treatment on the HRQoL and health status of patients in a real-world setting across a number of differing geographic regions.

## Methods

### Study design

Data were drawn from the Adelphi Gastric Cancer Disease Specific Programme (DSP)™, a point-in-time survey of clinical oncologists and gastroenterologists, and their consulting patients, conducted in China, France, Germany, Japan, the United Kingdom (UK) and the United States (US) between April and October 2019. To be invited to participate in the survey, clinical oncologists and gastroenterologists must have been practicing for more than five and less than 35 years and been involved in treatment decisions for a minimum of ten patients with GC/GEJC/EAC per typical calendar month. Sampling was conducted in a stratified random fashion within regions, with caps applied to reduce bias of oversampling at any given site or region, and to maximize representativeness of the sample.

Clinicians included in the survey were invited to recruit up to twelve consecutively consulting patients. To be eligible, patients had to be aged 18 years or over, have a clinician confirmed diagnosis of unresectable advanced or metastatic GC/GEJC/EAC, an Eastern Cooperative Oncology Group (ECOG) score of ≤ 2, and be receiving 1L active drug treatment (excluding clinical trials) or best supportive care (BSC) at point of consultation.

Patients were invited to complete, on a voluntary basis, two validated measures of disease activity and its impact on HRQoL and health status during the seven days prior to consultation. The EQ-5D-3L was used to measure overall quality of life and comprises two components, a 0-100 mm Visual Analogue Scale (VAS) that assesses the patient’s health and a descriptive utility index component which is made up of 5 dimensions (mobility, self-care, usual activities, pain/discomfort, and anxiety/depression), that are ranked on 3-level Likert scale where the patient can choose either, “no problems”, “some problems”, and “extreme problems” [13, 14]. The Functional Assessment of Cancer Therapy-General (FACT-G) questionnaire together with the Gastric Cancer module (FACT-Ga) [15] was used to measure HRQoL in cancer patients and is comprised of four domains: physical, social, emotional, and functional well-being. Patients must rate their status in each of these domains in the past seven days on a five-point Likert scale ranging from “not at all”, “a little bit”, “somewhat”, “quite a bit”, “very much” [16].

Patients completed their questionnaires independently from clinicians, returning them to the sponsor in sealed envelopes to ensure confidentiality. Upon return of the patient questionnaire, clinicians were required to provide anonymized data for each corresponding patient on an electronic case report form (eCRF) that included information on patient demographics, clinical characteristics, comorbidities, and symptoms, and 1L treatment received.

### Data analysis

The present analysis included only patients in a 1L treatment setting. The eCRFs were completed online to reduce the issue of missing data; however, for questions where the clinician may not have been certain of the answer, a response option of “don’t know” was included. Missing data in the patient questionnaire were not imputed, and the base (n) for each variable is reported, thereby enabling the calculation of number of patients excluded from analysis due to missing values. The analysis of the data was primarily descriptive in nature, with descriptive statistics depending on the type of variable being described. Categorical variables were described by counts and proportions of respondents, and continuous numerical variables were described by their means and standard deviations (SD), together with median and interquartile range (IQR).

The EQ-5D-3L utility index scores were calculated using the Time Trade-Off valuation model and applying the relevant tariff for each country. The EQ-5D-3L utility index scores and reference values for the EuroQol-Visual Analogue Scale (EQ-VAS) [17] were used for country comparison; minimally important differences for the EQ-5D-3L utility index score and EQ-VAS were 0.10 and 7, respectively [18]. Reference values from studies in patients with different forms of advanced cancer were also used for the FACT-G and its subdomains: Physical well-being (PWB), social/family well-being (SWB), emotional well-being (EWB), and functional well-being (FWB) [19].

Patients were stratified by human epidermal growth factor receptor 2 (HER2) testing status (positive, negative, or unknown) because HER2 has been identified as a potential therapeutic target. Up to 20% of patients with GC/GEJC/EAC have HER2 overexpression (HER2 positive) but patients with the more prevalent HER2 negative status have limited treatment options [20]. For the purposes of this study, we combined results from HER2 negative patients with patients with unknown HER2 status, as at the time of data collection there was no specific treatment options for HER2 negative patients.

Regression analysis was used to explore the drivers of treatment selection (1L vs BSC; logistic regression), and the drivers of HRQoL, as measured by the EQ-5D-3L index, EQ-VAS, FACT-Ga, and FACT-G. Initially, Least Absolute Shrinkage and Selection Operator (LASSO) regression models [21] with cross validation were used to select predictors to include in the final models from the following variables (forced predictors): age, diagnosis (GC vs GEJC vs EAC), ECOG status at advanced diagnosis, whether ECOG status had changed from diagnosis to data collection, Tumor, Nodes, Metastases (TNM) stage at advanced diagnosis, de novo vs recurrent disease, time since advanced diagnosis, insurance (insurance vs no insurance; for active 1L vs BSC only). The models also included sex, Body Mass Index (BMI), smoking status, alcohol status, Charlson Comorbidity Index (CCI) [22, 23], time since initial diagnosis, Lauren classification, non-drug treatments (surgery, radiotherapy, stent, laser therapy), symptom groups (gastrointestinal, respiratory, sickness and other symptoms), and insurance (insurance vs no insurance, for HRQoL instruments only), with these predictors only being including in the following regression models if they had a non-zero coefficient in the LASSO model. To test the impact of disease occurrence, either de novo or recurrent disease, on each dimension of the EQ-5D-3L utility index we performed bivariate analysis involving hypothesis testing using chi-squared tests. To test the effect of drinking level (“regular heavy drinker”, “binge drinker”, “regular drinker”, “occasional drinker”, “frequent drinker”, “non-drinker/abstinent”) on FACT-Ga scores, we used one-way analysis of variance (ANOVA) tests.

## Results

A total of 995 patients were recruited from 284 clinicians in China (200; 20.1%), France (203; 20.4%), Germany (200; 20.1%), Japan (45; 4.5%), the UK (204; 20.5%), and the US (143; 14.4%). Demographic characteristics were similar across all countries; most patients (69%) were male and had a mean (SD) age of 65.0 (10.3) years. The majority of patients had a confirmed diagnosis of GC (61%), 27% of GEJC, and 12% of EAC; China had the lowest proportions of GEJC (14%) and EAC patients (1%). Of the total 995 patients, 21% tested HER2 positive, with the remainder (78.9%) either HER2 negative or unknown (results inconclusive or untested). Most patients (87%) were on active treatment; of these patients (*n* = 870), most received dual (44%) or triple (29%) chemotherapy, with 15% of patients receiving anti-HER2 based therapy, and only a very small proportion (3%) receiving immunotherapy (Table 1).

**Table 1:** Demographics, clinical characteristics, and treatment regimens of patients with GC/GEJC/EAC

Characteristic	Total	France	Germany	UK	US	Japan	China
	***n* = 995**	***n* = 203**	***n* = 200**	***n* = 204**	***n* = 143**	***n* = 45**	***n* = 200**
**Age, years**							
Mean (SD)	65.0 (10.3)	65.0 (9.7)	65.0 (9.5)	67.7 (9.7)	67.9 (9.2)	70.2 (8.6)	59.0 (10.9)
Median (IQR)	66.0 (59–72)	67.0 (60–71)	65.0 (58–71)	68.5 (64–74)	68.0 (61–73)	70.0 (64–78)	60.0 (51–67)
**Male, n (%)**	691 (69.5)	150 (73.9)	146 (73.0)	132 (64.7)	104 (72.7)	28 (62.2)	131 (65.5)
**Female, n (%)**	304 (30.5)	53 (26.1)	54 (27.0)	72 (35.3)	39 (27.3)	17 (37.8)	69 (34.5)
**Confirmed diagnosis, n (%)**	**995**	**203**	**200**	**204**	**143**	**45**	**200**
GC	610 (61.3)	130 (64.0)	121 (60.5)	100 (49.0)	57 (39.9)	31 (68.9)	171 (85.5)
GEJC	268 (26.9)	54 (26.7)	59 (29.5)	64 (31.4)	56 (39.2)	8 (17.8)	27 (13.5)
EAC	117 (11.8)	19 (9.4)	20 (10.0)	40 (19.6)	30 (21.0)	6 (13.3)	2 (1.0)
**Lauren classification, n (%)**	**873**	**181**	**195**	**170**	**100**	**39**	**188**
Diffuse	340 (39.0)	68 (37.6)	72 (36.9)	75 (44.1)	38 (38.0)	19 (48.7)	68 (36.2)
Intestinal	375 (43.0)	89 (49.2)	85 (43.6)	61 (35.9)	38 (38.0)	18 (46.3)	84 (44.7)
Mixed	158 (18.1)	24 (13.3)	38 (19.5)	34 (20.0)	24 (24.0)	2 (5.1)	36 (19.2)
**HER2 status, n (%)**	**995**	**203**	**200**	**204**	**143**	**45**	**200**
HER2 positive	210 (21.1)	30 (14.8)	42 (21.0)	27 (13.2)	20 (14.0)	5 (11.1)	86 (43.0)
HER2 status negative or unknown	785 (78.9)	173 (85.2)	158 (79.0)	177 (86.8)	123 (86.0)	40 (88.9)	114 (57.0)
**Current therapy, n (%)**	**995**	**203**	**200**	**204**	**143**	**45**	**200**
Active first-line systemic therapy	870 (87.4)	198 (98.0)	183 (91.5)	172 (84.3)	118 (82.5)	41 (91.1)	158 (79.0)
Best Supportive Care only	125 (12.6)	5 (2.5)	17 (8.5)	32 (15.7)	25 (17.5)	4 (8.9)	42 (21.0)
**Current active treatment regimen, n (%)**	**870**	**198**	**183**	**172**	**118**	**41**	**158**
Anti-angiogenic based	28 (3.2)	0 (0.0)	14 (7.7)	0 (0.0)	4 (3.4)	2 (4.9)	8 (5.1)
Mono chemotherapy	42 (4.8)	6 (3.0)	1 (0.6)	5 (2.9)	9 (7.6)	12 (29.3)	9 (5.7)
Dual chemotherapy	383 (44.0)	111 (56.1)	51 (27.9)	67 (39.0)	59 (50.0)	21 (51.2)	74 (46.8)
Triple chemotherapy	254 (29.2)	49 (24.8)	84 (45.9)	75 (43.6)	17 (14.4)	1 (2.4)	28 (17.7)
Anti-HER2 based	134 (15.4)	31 (15.7)	33 (18.0)	25 (14.5)	10 (8.5)	3 (7.3)	32 (20.3)
Anti-PD1 IO	29 (3.3)	1 (0.5)	0 (0.0)	0 (0.0)	19 (16.1)	2 (4.9)	7 (4.4)

*EAC* Esophageal adenocarcinoma, *GC* Gastric cancer, *GEJC* Gastroesophageal junction cancer, *HER2* Human epidermal growth factor receptor 2, *IQR* Interquartile range, *SD* Standard deviation, *UK* United Kingdom, *US* United States, *PD1 IO* Programmed cell death protein 1 immuno-oncology

Of the 682 patients (68.5% of the total sample) whose HER2 status was negative or unknown and were receiving active 1L treatment, the mean (SD) age was 64.6 (9.5) years, 70% were male, and 61% were diagnosed with GC, 28% with GEJC, and 11% with EAC. On average, patients in China were the youngest and patients in Japan the eldest (56 years vs 70 years, respectively). Most patients had intestinal Lauren classification (44%), with similar proportions across all countries, with the exception of patients from the UK and Japan, who mainly had diffuse Lauren classification. Patients had a mean (SD) CCI score of 2.7 (1.3), indicating some comorbidity burden, the most frequent comorbidities being diabetes, pulmonary disease, anxiety, and depression. Dual and triple therapies were the most commonly used 1L treatment (51% and 35%, respectively); interestingly, 1% of patients received anti-HER2 therapy, while 18% of patients in the US also received anti-programmed cell death protein 1 (anti-PD1) immunotherapy, compared with < 6% in other countries (Table 2).

**Table 2:** Demographics, clinical characteristics, and treatments of GC/GEJC/EAC patients, HER2 negative/ unknown, on first-line treatment

Characteristic	Total	France	Germany	UK	US	Japan	China
	***n* = 682**	***n* = 168**	***n* = 146**	***n* = 145**	***n* = 99**	***n* = 36**	***n* = 88**
**Age, years**							
Mean (SD)	64.6 (10.0)	65.0 (9.5)	64.1 (8.9)	66.3 (8.9)	67.7 (8.6)	69.8 (8.3)	56.1 (11.4)
Median (IQR)	66 (59–71)	67 (60–71)	65 (58–69)	67 (63–72)	68 (62–73)	70 (63–77)	57 (48–65)
**Male (%)**	474 (69.5)	127 (75.6)	108 (74.0)	95 (65.5)	70 (70.7)	23 (63.9)	51 (58.0)
**Confirmed diagnosis, n (%)**	**682**	**168**	**146**	**145**	**99**	**36**	**88**
GC	415 (60.9)	107 (63.7)	90 (61.6)	75 (51.7)	41 (41.4)	26 (72.2)	76 (86.4)
GEJC	192 (28.2)	44 (26.2)	43 (29.5)	46 (31.7)	43 (43.4)	4 (11.1)	12 (13.6)
EAC	75 (11.0)	17 (10.1)	13 (8.9)	24 (16.6)	15 (15.2)	6 (16.7)	0 (0.0)
**Lauren classification, n (%)**	**599**	**148**	**144**	**126**	**68**	**31**	**82**
Diffuse	231 (38.6)	57 (38.5)	52 (36.1)	52 (41.3)	26 (38.2)	16 (51.6)	28 (34.2)
Intestinal	262 (43.7)	73 (49.3)	64 (44.4)	47 (37.3)	30 (44.1)	13 (41.9)	35 (42.7)
Mixed	106 (17.7)	18 (12.2)	28 (19.4)	27 (21.4)	12 (17.7)	2 (6.5)	19 (23.2)
**Comorbidities (> 5%), n (%)**	**682**	**168**	**146**	**145**	**99**	**36**	**88**
Diabetes	107 (15.7)	22 (13.1)	24 (16.4)	22 (15.3)	22 (22.2)	3 (8.3)	14 (15.9)
Chronic pulmonary disease	96 (14.1)	24 (14.3)	30 (20.6)	22 (15.3)	15 (15.2)	0 (0.0)	5 (5.7)
Anxiety	82 (12.0)	35 (20.8)	8 (5.5)	8 (5.5)	19 (19.2)	2 (5.6)	10 (11.5)
Depression	80 (11.7)	15 (8.9)	27 (18.5)	12 (8.3)	23 (23.2)	0 (0.0)	3 (3.4)
Peptic ulcer disease	68 (10.0)	20 (11.9)	3 (2.1)	18 (12.4)	14 (14.1)	2 (5.6)	11 (12.5)
Mild liver disease	56 (8.2)	12 (7.1)	17 (11.6)	3 (2.1)	8 (8.1)	7 (19.4)	9 (10.2)
Peripheral vascular disease	39 (5.7)	15 (8.9)	9 (6.2)	2 (1.4)	10 (10.1)	0 (0.0)	3 (3.4)
Myocardial infarction	37 (5.4)	8 (4.8)	13 (8.9)	9 (6.2)	5 (5.1)	0 (0.0)	2 (2.3)
Renal disease	34 (5.0)	7 (4.2)	7 (4.8)	6 (4.1)	8 (8.1)	2 (5.6)	4 (4.6)
**Charlson Comorbidity Index**	**682**	**168**	**146**	**145**	**99**	**36**	**88**
Mean (SD)	2.7 (1.3)	2.6 (1.1)	3.0 (1.6)	2.7 (1.3)	2.7 (1.3)	2.8 (1.2)	2.5 (1.1)
Median (IQR)	2 (2–3)	2 (2–3)	2 (2–3)	2 (2–3)	2 (2–3)	2 (2–4)	2 (2–3)
**Current active treatment regimen, n (%)**	**682**	**168**	**146**	**145**	**99**	**36**	**88**
Anti-angiogenic based	22 (3.2)	0 (0.0)	11 (7.5)	0 (0.0)	4 (4.0)	1 (2.8)	6 (6.8)
Mono chemotherapy	37 (5.4)	6 (3.6)	1 (0.7)	5 (3.5)	9 (9.1)	11 (30.6)	5 (5.7)
Dual chemotherapy	350 (51.3)	111 (66.1)	50 (34.3)	66 (45.5)	57 (57.6)	21 (58.3)	45 (51.1)
Triple chemotherapy	238 (34.9)	48 (28.6)	82 (56.2)	74 (51.0)	11 (11.1)	1 (2.8)	22 (25.0)
Anti-HER2 based	9 (1.3)	2 (1.2)	2 (1.4)	0 (0.0)	0 (0.0)	0 (0.0)	5 (5.8)
Anti-PD1 IO	26 (3.8)	1 (0.6)	0 (0.0)	0 (0.0)	18 (18.2)	2 (5.6)	5 (5.7)

*EAC* Esophageal adenocarcinoma, *GC* Gastric cancer, *GEJC* Gastroesophageal junction cancer, *HER2* Human epidermal growth factor receptor 2, *IQR* Interquartile range, *PD1 IO* Programmed cell death protein 1 immuno-oncology, *SD* Standard deviation, *UK* United Kingdom, *US* United States

The median time since initial diagnosis of GC/GEJC/EAC to data collection in this subgroup was 3 months (IQR: 2–6 months), and 3 months (IQR: 2–5) since an advanced diagnosis, although a longer median time since advanced diagnosis was observed in Japan (6 months; IQR: 2–11 months). At advanced diagnosis, most patients (62%) had an ECOG status of 1, with 76% of patients’ ECOG status remaining unchanged at data collection, while the majority of patients were at TNM stage IV, both at advanced diagnosis and at data collection (67% and 73%, respectively). On average, 81% of patients had de novo disease, ranging from 42% in China to 95% in the UK (Table 3). At the time of data collection, these patients had been on 1L treatment for an average of 2 months, with the majority achieving stable disease or partial response (94%). Half of the patients had also received nonpharmacological treatment (surgery: 24%; radiotherapy: 15%; laser therapy: 11%). A total of 383 (57%) patients reported being ‘somewhat, quite a bit, or very much’ bothered by the side effects of their treatment (Table 4).

**Table 3:** Clinical characteristics of patients with GC/GEJC/EAC, HER2 status negative/unknown, on first-line treatment

Disease status	Total	France	Germany	UK	US	Japan	China
	***n* = 682**	***n* = 168**	***n* = 146**	***n* = 145**	***n* = 99**	***n* = 36**	***n* = 88**
**Time since initial diagnosis, months**	**625**	**156**	**135**	**129**	**86**	**31**	**88**
Mean (SD)	6.3 (13.0)	6.2 (10.0)	5.3 (6.2)	3.3 (3.4)	6.4 (13.2)	10.3 (12.4)	11.3 (26.3)
Median (IQR)	3 (2–6)	3 (2–5)	3 (2–5)	2 (2–4)	3 (2–5)	6 (2–11)	6 (3–14)
**Time since advanced diagnosis, months**	**629**	**155**	**139**	**129**	**87**	**31**	**88**
Mean (SD)	4.8 (7.6)	4.5 (7.1)	4.8 (5.6)	3.1 (3.1)	6.0 (13.1)	8.5 (9.6)	5.0 (7.1)
Median (IQR)	3 (2–5)	3 (2–4)	3 (2–5)	2 (2–3)	3 (2–5)	6 (2–10)	3 (2–5)
**ECOG at advanced diagnosis, n (%)**	**679**	**168**	**143**	**145**	**99**	**36**	**88**
0	98 (14.4)	21 (12.5)	8 (5.6)	31 (21.4)	11 (11.1)	15 (41.7)	12 (13.6)
1	421 (62.0)	101 (60.1)	100 (69.9)	102 (70.3)	58 (58.6)	15 (41.7)	45 (51.1)
2	152 (22.4)	45 (26.8)	35 (24.5)	12 (8.3)	23 (23.2)	6 (16.7)	31 (35.2)
**Current ECOG**^**a**^**, n (%)**	**682**	**168**	**146**	**145**	**99**	**36**	**88**
0	73 (10.7)	13 (7.7)	6 (4.1)	27 (18.6)	12 (12.1)	9 (25.0)	6 (6.8)
1	388 (56.9)	92 (54.9)	76 (52.1)	105 (72.4)	58 (58.6)	19 (52.8)	38 (43.2)
2	221 (32.4)	63 (37.5)	64 (43.8)	13 (9.0)	29 (29.3)	8 (22.2)	44 (50.0)
**ECOG change**^**b**^**, n (%)**	**672**	**167**	**138**	**145**	**99**	**36**	**87**
Unchanged	510 (75.9)	132 (79.0)	96 (69.6)	129 (89.0)	80 (80.8)	24 (66.7)	49 (56.3)
Changed	162 (24.1)	35 (21.0)	42 (30.4)	16 (11.0)	19 (19.2)	12 (33.3)	38 (43.7)
**TNM staging at advanced diagnosis, n (%)**	**682**	**168**	**146**	**145**	**99**	**36**	**88**
Stage IIIA	74 (10.9)	6 (3.6)	28 (19.2)	3 (2.1)	10 (10.1)	1 (2.8)	26 (29.6)
Stage IIIB	79 (11.6)	8 (4.8)	28 (19.2)	2 (1.4)	9 (9.1)	1 (2.8)	31 (35.2)
Stage IIIC	72 (10.6)	19 (11.3)	16 (11.0)	12 (8.3)	11 (11.1)	0 (0.0)	14 (15.9)
Stage IV	457 (67.0)	135 (80.4)	74 (50.7)	128 (88.3)	69 (69.7)	34 (94.4)	17 (19.3)
**Current TNM staging, n (%)**	**682**	**168**	**146**	**145**	**99**	**36**	**88**
Stage IIIA	37 (5.4)	1 (0.6)	15 (10.3)	1 (0.7)	8 (8.1)	0 (0.0)	12 (13.6)
Stage IIIB	68 (10.0)	8 (4.8)	21 (14.4)	1 (0.7)	8 (8.1)	0 (0.0)	30 (34.1)
Stage IIIC	78 (11.4)	19 (11.3)	15 (10.3)	11 (7.6)	10 (10.1)	1 (2.8)	22 (25.0)
Stage IV	499 (73.2)	140 (83.3)	95 (65.1)	132 (91.0)	73 (73.7)	35 (97.2)	24 (27.3)
**De novo vs recurrent disease, n (%)**	**682**	**168**	**146**	**145**	**99**	**36**	**88**
De novo	552 (80.9)	151 (89.9)	107 (73.3)	138 (95.2)	87 (87.9)	32 (88.9)	37 (42.1)
Recurrent	130 (19.1)	17 (10.1)	39 (26.7)	7 (4.8)	12 (12.1)	4 (11.1)	51 (58.0)

*EAC* Esophageal adenocarcinoma, *ECOG* Eastern cooperative oncology group, *GC* Gastric cancer, *GEJC* Gastroesophageal junction cancer, *HER2* Human epidermal growth factor receptor 2, *IQR* Interquartile range, *SD* Standard deviation, *TNM* Tumor, Nodes, Metastases, *UK* United Kingdom, *US* United States

^a^no patients had an ECOG status of 3 or 4

^b^relating to change in ECOG status from advanced diagnosis to current

**Table 4:** Treatment effects in patients with GC/GEJC/EAC, with negative/unknown HER2, on first-line treatment

Observations	Total *n* = 682	France *n* = 168	Germany *n* = 146	UK *n* = 145	US *n* = 99	Japan *n* = 36	China *n* = 88
**Time since start of first-line treatment, days**	**629**	**158**	**139**	**123**	**88**	**35**	**86**
Mean (SD)	90 (163)	80 (93)	78 (86)	62 (43)	125 (370)	177 (193)	95 (69)
Median (IQR)	62 (30–103)	51 (26–99)	62 (30–987)	49 (30–85)	58 (30–103)	166 (30–250)	81 (50–122)
**Response to current therapy, n (%)**	**469**	**86**	**112**	**84**	**73**	**30**	**84**
Stable disease	166 (35.4)	32 (37.2)	41 (36.6)	25 (29.8)	25 (34.3)	16 (53.3)	27 (32.1)
Partial response	273 (58.2)	49 (57.0)	67 (59.8)	59 (70.2)	38 (52.1)	10 (33.3)	50 (59.5)
Complete response	8 (1.7)	2 (2.3)	0 (0.0)	0 (0.0)	4 (5.5)	0 (0.0)	2 (2.4)
Disease progression	22 (4.7)	3 (3.5)	4 (3.6)	0 (0.0)	6 (8.2)	4 (13.3)	5 (6.0)
**Nonpharmacological treatments, n (%)**	**682**	**168**	**146**	**145**	**99**	**36**	**88**
Surgery	162 (23.8)	19 (11.3)	65 (44.5)	11 (7.6)	17 (17.2)	7 (19.4)	43 (48.9)
Radiotherapy	103 (15.1)	17 (10.1)	35 (24.0)	10 (6.9)	22 (22.2)	1 (2.8)	18 (20.5)
Stent	72 (10.6)	16 (9.5)	12 (8.2)	30 (20.7)	10 (10.1)	3 (8.3)	1 (1.1)
Laser therapy	5 (0.7)	0 (0.0)	4 (2.7)	0 (0.0)	1 (1.0)	0 (0.0)	0 (0.0)
None	5 (0.7)	0 (0.0)	4 (2.7)	0 (0.0)	1 (1.0)	0 (0.0)	0 (0.0)
**Bothered by side effects, n (%)**	**676**	**166**	**144**	**145**	**98**	**35**	**88**
Not at all	81 (12.0)	8 (4.8)	10 (6.9)	21 (14.5)	22 (22.5)	2 (5.7)	18 (20.5)
A little bit	212 (31.4)	62 (37.4)	42 (29.2)	48 (33.1)	27 (27.6)	15 (42.9)	18 (20.5)
Somewhat	247 (36.5)	51 (30.7)	67 (46.5)	55 (37.9)	25 (25.5)	14 (40.0)	35 (39.8)
Quite a bit	110 (16.3)	42 (25.3)	19 (13.2)	14 (9.7)	19 (19.4)	3 (8.6)	13 (14.8)
Very much	26 (3.9)	3 (1.8)	6 (4.2)	7 (4.8)	5 (5.1)	1 (2.9)	4 (4.6)

*EAC* Esophageal adenocarcinoma, *GC* Gastric cancer, *GEJC* Gastroesophageal junction cancer, *HER2* Human epidermal growth factor receptor 2, *IQR* Interquartile range, *SD* Standard deviation, *UK* United Kingdom, *US* United States

**Fig. 1:**
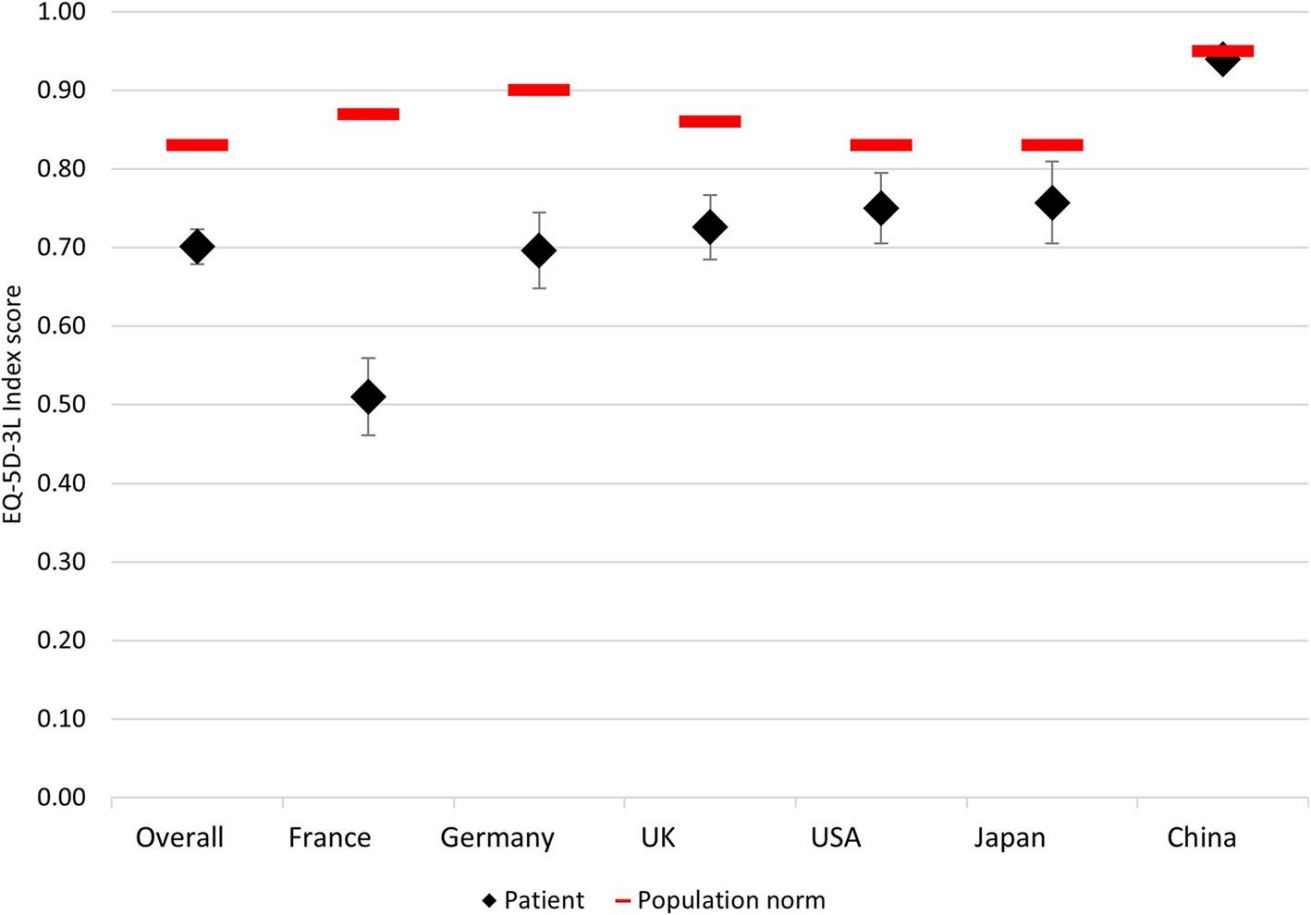
EQ-5D-3L utility index scores for patients with GC/GEJC/EAC, HER2 status negative/unknown, on first-line treatment. EAC, esophageal adenocarcinoma; EQ-5D-3L, EuroQol 5-Dimension 3-Level Questionnaire; GC, gastric cancer; GEJC, gastroesophageal junction cancer; UK, United Kingdom; US, United States

**Fig. 2:**
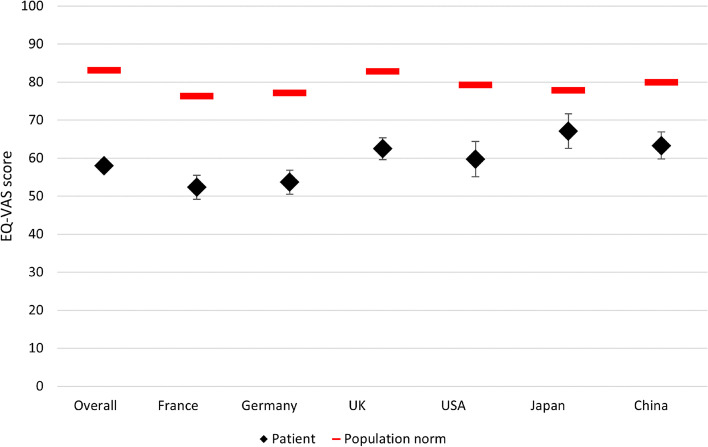
EQ-VAS scores for patients with GC/GEJC/EAC, HER2 status negative/unknown, on first-line treatment. EAC, esophageal adenocarcinoma; EQ-VAS, EuroQol-Visual Analogue Scale; GC, gastric cancer; GEJC, gastroesophageal junction cancer; UK, United Kingdom; US, United States

**Fig. 3:**
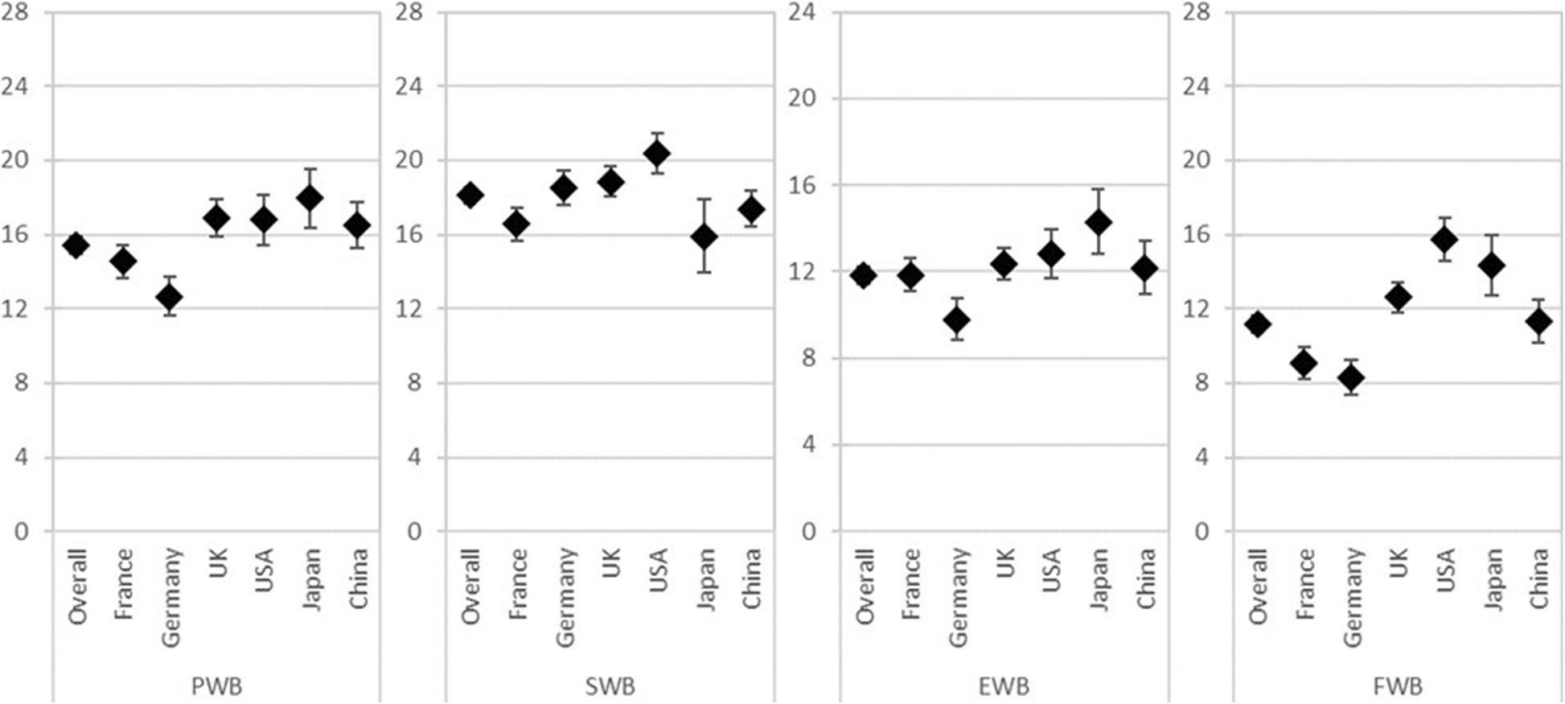
FACT-G subscales for patients with GC/GEJC/EAC, HER2 status negative/unknown, on first-line treatment. EAC, esophageal adenocarcinoma; EWB, emotional well-being (range 0–24); FACT-G, Functional Assessment of Cancer Therapy-General; FWB, functional well-being (range 0–28); GC, gastric cancer; GEJC, gastroesophageal junction cancer; PWB, physical well-being (range 0–28); SWB, social well-being (range 0–28); UK, United Kingdom; US, United States

The subgroup of patients with HER2 status negative or unknown and receiving active 1L treatment reported a mean (SD) health status score of 0.701 (0.285) as measured by the EQ-5D-3L utility index. Patients in France, Germany, the UK, and the US reported overall worse health status compared to the general population (*p* < 0.001) [17] (Table 5, Fig. 1). The overall EQ-VAS score of 58.1 was also lower than the general population norm (83.1) [24], indicating a significant impairment in health (Table 5, Fig. 2). With a similar distribution across all considered countries, the subscale scores on the FACT questionnaire indicated impaired HRQoL in GC/GEJC/EAC patients (Fig. 3), with overall mean scores on the FACT-G and FACT-Ga also showing diminished HRQoL compared to norm values (56.9 vs 85.2 [*p* < 0.001] and 100.4 vs 144.7 [*p* = 0.8400], respectively) [15, 25]. Finally, an overall mean score of 54.1 on the Katz Index indicated some impairment in activities of daily living, which was supported by reported interference of their illness in both daily activities and social life (Table 5).

**Table 5:** Impact ofGC/GEJC/EAC on patient-reported outcomes in patients with negative/unknown HER2, on first-line treatment

	**Total**	**France**	**Germany**	**UK**	**US**	**Japan**	**China**
**EQ-5D-3L utility index**	**672**	**161**	**143**	**145**	**99**	**36**	**88**
Mean (SD)	0.701 (0.285)	0.510 (0.317)	0.696 (0.290)	0.726 (0.255)	0.750 (0.222)	0.757 (0.153)	0.940 (0.060)
Median (IQR)	0.779 (0.585–0.928)	0.510 (0.275–0.798)	0.788 (0.564–0.887)	0.744 (0.620–0.848)	0.781 (0.594–1.000)	0.714 (0.672–0.804)	0.946 (0.923–0.973)
**EQ-VAS**	**675**	**167**	**143**	**144**	**98**	**35**	**88**
Mean (SD)	58.1 (19.8)	52.3 (20.6)	53.7 (19.1)	62.5 (17.3)	59.7 (23.1)	67.1 (13.4)	63.3 (16.7)
Median (IQR)	60 (45–75)	50 (35–70)	55 (40–70)	65 (50–77)	60 (40–80)	70 (60–76)	65 (56–75)
**FACT: Physical well-being**	**679**	**166**	**146**	**145**	**99**	**35**	**88**
Mean (SD)	15.4 (6.3)	14.5 (5.6)	12.7 (6.4)	16.9 (5.9)	16.8 (6.8)	18.0 (4.7)	16.5 (6.0)
Median (IQR)	15 (11–20)	14 (11–19)	13 (8–17)	17 (14–21)	17 (11–23)	18 (15–21)	17 (12–21)
**FACT: Social/family well-being**	**678**	**167**	**144**	**145**	**99**	**35**	**88**
Mean (SD)	18.1 (5.5)	16.6 (5.7)	18.5 (5.7)	18.9 (5.1)	20.4 (5.4)	15.9 (5.8)	17.4 (4.5)
Median (IQR)	19 (13–22)	17 (12–21)	19 (15–22)	19 (16–22)	21 (17–25)	17 (11–21)	18 (14–20)
**FACT: Emotional well-being**	**679**	**167**	**146**	**145**	**97**	**36**	**88**
Mean (SD)	11.8 (5.3)	11.9 (5.2)	9.8 (5.7)	12.3 (4.4)	12.8 (5.6)	14.3 (4.4)	12.2 (5.7)
Median (IQR)	12 (8–15)	12 (9–15)	10 (5–14)	12 (10–15)	13 (8–18)	15 (10–17)	11 (8–17)
**FACT: Functional well-being**	**680**	**168**	**146**	**145**	**97**	**36**	**88**
Mean (SD)	11.2 (6.0)	9.1 (5.6)	8.3 (5.6)	12.6 (4.9)	15.7 (5.8)	14.4 (4.8)	11.3 (5.4)
Median (IQR)	11 (7–15)	9 (5–13)	8 (3–12)	12 (9–16)	16 (13–20)	14 (10–18)	12 (8–14)
**FACT-Ga**	**677**	**167**	**145**	**145**	**96**	**36**	**88**
Mean (SD)	40.9 (14.2)	39.5 (13.3)	35.5 (14.2)	44.2 (13.6)	44.3 (15.0)	44.6 (10.5)	42.1 (14.6)
Median (IQR)	41 (31–51)	40 (32–48)	35 (26–44)	45 (36–53)	43 (32–58)	44 (37–51)	40 (30–56)
**FACT: Trial outcome index**	**602**	**134**	**132**	**130**	**87**	**32**	**87**
Mean (SD)	68.0 (23.6)	63.8 (20.6)	57.1 (24.1)	73.8 (22.0)	77.8 (25.5)	75.2 (15.7)	69.8 (22.6)
Median (IQR)	68 (53–84)	65 (53–76)	60 (40–73)	74 (62–88)	75 (58–101)	75 (64–83)	69 (54–86)
**FACT-G**	296	67	43	70	49	18	49
Mean (SD)	56.94 (16.8)	51 (13.3)	49.9 (15.2)	61.3 (18.1)	62.9 (18.2)	58 (10.0)	58.6 (17.1)
Median (IQR)	54 (47–67)	50 (46–57)	50 (42–59)	61 (49–74)	59 (49–79)	56 (50–66)	57 (45–69)
**FACT: Gastric total**	**281**	**58**	**41**	**69**	**47**	**18**	**48**
Mean (SD)	100.4 (28.9)	94.4 (23.6)	89.0 (28.6)	106.9 (31.0)	106.0 (30.4)	99.8 (17.7)	102.4 (30.8)
Median (IQR)	97 (82–121)	93 (82–102)	94 (67–108)	103 (88–126)	99 (84–129)	101 (84–116)	98 (79–129)
**Katz index**	**611**	**151**	**138**	**118**	**81**	**35**	**88**
Mean (SD)	5.1 (1.5)	5.3 (1.2)	4.1 (1.9)	5.5 (1.0)	5.5 (1.2)	5.8 (0.6)	5.3 (1.1)
Median (IQR)	6 (5–6)	6 (5–6)	4 (3–6)	6 (5–6)	6 (6–6)	6 (6–6)	6 (5–6)
**Interference with daily activities**	**682**	**168**	**146**	**145**	**99**	**36**	**88**
Mean (SD)	4.2 (1.5)	4.6 (1.5)	4.8 (1.4)	3.8 (1.4)	4.0 (1.6)	3.8 (1.6)	3.4 (11.3)
Median (IQR)	4 (3–5)	5 (3–6)	5 (4–6)	4 (3–5)	4 (2–5)	4 (2–5)	4 (3–4)
**Interference with social life**	**682**	**168**	**146**	**145**	**99**	**36**	**88**
Mean (SD)	4.3 (1.5)	4.6 (1.4)	4.8 (1.5)	3.9 (1.4)	3.8 (1.6)	3.9 (1.5)	4.1 (1.6)
Median (IQR)	4 (3–5)	5 (4–6)	5 (4–6)	4 (3–5)	4 (2–5)	4 (2–5)	4 (3–5)

*EAC* Esophageal adenocarcinoma, *EQ-5D-3L* EuroQol 5-Dimension 3-Level questionnaire, *EQ-VAS* EuroQol-Visual Analogue Scale, *FACT* Functional Assessment of Cancer Therapy, *FACT-G* Functional Assessment of Cancer Therapy-General, *FACT-Ga* Functional Assessment of Cancer Therapy-Gastric Cancer, *GC* Gastric cancer, *GEJC* Gastroesophageal junction cancer, *HER2* Human epidermal growth factor receptor 2, *IQR* Interquartile range, *SD* Standard deviation, *UK* United Kingdom, *US* United States

Of the 682 patients with HER2 status negative or unknown, 81% were diagnosed de novo with advanced disease, whilst 19% had recurrent disease (Table 3). The demographic characteristics of the two subgroups shown in Table 6 indicate a slightly higher proportion of patients with GC having recurrence (70%). Accordingly, more patients with recurrent disease had undergone surgery or radiotherapy, and a larger proportion of de novo patients reported side effects of their treatment. Patient-reported HRQoL appeared to be worse in patients with recurrent disease, as illustrated by lower scores on the FACT questionnaires and the EQ-VAS (Table 6).

**Table 6:** Demographics, clinical characteristics, and treatments of GC/GEJC/EAC patients according to de novo or recurrent disease

	**Total**	**De novo**	**Recurrent**
**Age, years**	**682**	**552**	**130**
Mean (SD)	64.6 (10.0)	65.6 (9.8)	60.3 (9.6)
Median (IQR)	66 (59–71)	67 (60–72)	62 (55–68)
**Confirmed diagnosis, n (%)**	**682**	**552**	**130**
GC	415 (60.9)	324 (58.7)	91 (70.0)
GEJC	192 (28.2)	162 (29.4)	30 (23.1)
EAC	75 (11.0)	66 (12.0)	9 (6.9)
**Time since diagnosis, months**	**625**	**502**	**123**
Mean (SD)	6.3 (13.0)	3.6 (4.7)	17.6 (24.8)
Median (IQR)	3 (2–6)	2 (2–4)	13 (7–20)
**Time since advanced diagnosis, months**	**629**	**503**	**126**
Mean (SD)	4.8 (7.6)	3.6 (4.7)	9.5 (13.2)
Median (IQR)	3 (2–5)	3 (2–4)	5 (3–11)
**Nonpharmacological treatments, n (%)**	**682**	**552**	**130**
Surgery	162 (23.8)	75 (13.6)	87 (66.9)
Radiotherapy	103 (15.1)	70 (12.7)	33 (25.8)
Stent	72 (10.6)	61 (11.1)	11 (8.5)
Laser therapy	5 (0.7)	5 (0.9)	0 (0.0)
None	400 (58.7)	382 (69.2)	18 (13.9)
**Side effect experienced (> 10%), n (%)**	**296 (45.0)**	**210 (39.3)**	**86 (69.4)**
Nausea	186 (62.8)	128 (61.0)	58 (67.4)
Fatigue	144 (48.7)	98 (46.7)	46 (53.5)
Anemia	106 (35.8)	72 (34.3)	34 (39.5)
Loss of appetite	103 (34.8)	62 (29.5)	41 (47.7)
Vomiting	97 (32.8)	52 (24.8)	45 (52.3)
Low white blood cell count	86 (29.1)	59 (28.1)	27 (31.4)
Diarrhea	81 (27.4)	61 (29.1)	20 (23.3)
Weight loss/gain	66 (22.3)	35 (16.7)	31 (36.1)
Low platelet count	59 (19.9)	38 (18.1)	21 (24.4)
Numbness in hands and feet	56 (18.9)	38 (18.1)	18 (20.93)
Hair loss/thinning	54 (18.2)	27 (12.9)	27 (31.4)
Abdominal pain	30 (10.1)	15 (7.1)	15 (17.4)
**Bothered by side effects, n (%)**	**676**	**550**	**126**
Not at all	81 (12.0)	68 (12.4)	13 (10.3)
A little bit	212 (31.4)	180 (32.7)	32 (25.4)
Somewhat	247 (36.5)	191 (34.7)	56 (44.4)
Quite a bit	110 (16.3)	89 (16.2)	21 (16.7)
Very much	26 (3.5)	22 (4.0)	4 (3.2)
**EQ-5D-3L utility index**	**672**	**544**	**128**
Mean (SD)	0.70 (0.29)	0.69 (0.29)	0.77 (0.24)
Median (IQR)	0.78 (0.59–0.93)	0.76 (0.55–0.89)	0.89 (0.70–0.94)
**EQ-VAS**	**675**	**550**	**125**
Mean (SD)	58.1 (19.8)	58.4 (20.2)	56.6 (18.1)
Median (IQR)	60 (45–75)	60 (45–75)	60 (47–70)
**FACT: Gastric cancer subscale**	**677**	**549**	**128**
Mean (SD)	40.9 (14.2)	41.4 (14.5)	39.0 (12.6)
Median (IQR)	41 (31–51)	41 (32–52)	38 (31–48)
**FACT: Trial outcome index**	**602**	**489**	**113**
Mean (SD)	68.0 (23.6)	68.8 (24.4)	64.4 (19.4)
Median (IQR)	68 (53–84)	69 (54–86)	65 (53–77)
**FACT-G**	**296**	**244**	**52**
Mean (SD)	56.9 (16.8)	57.5 (17.5)	54.4 (13.0)
Median (IQR)	54 (47–67)	55 (48–69)	53 (45–62)
**FACT: Gastric total**	**281**	**230**	**51**
Mean (SD)	100.3 (28.9)	101.2 (30.1)	96.4 (22.6)
Median (IQR)	97 (82–121)	98 (83–123)	97 (81–111)

*EAC* Esophageal adenocarcinoma, *EQ-5D-3L* EuroQol 5-Dimension 3-Level questionnaire, *EQ-VAS* EuroQol—Visual Analogue Scale, *FACT* Functional Assessment of Cancer Therapy, *FACT-G* Functional Assessment of Cancer Therapy – General, *FACT-Ga* Functional Assessment of Cancer Therapy—Gastric Cancer, *GC* Gastric cancer, *GEJC* Gastroesophageal junction cancer, *HER2* Human epidermal growth factor receptor 2, *IQR* Interquartile range, *SD* Standard deviation

**Fig. 4:**
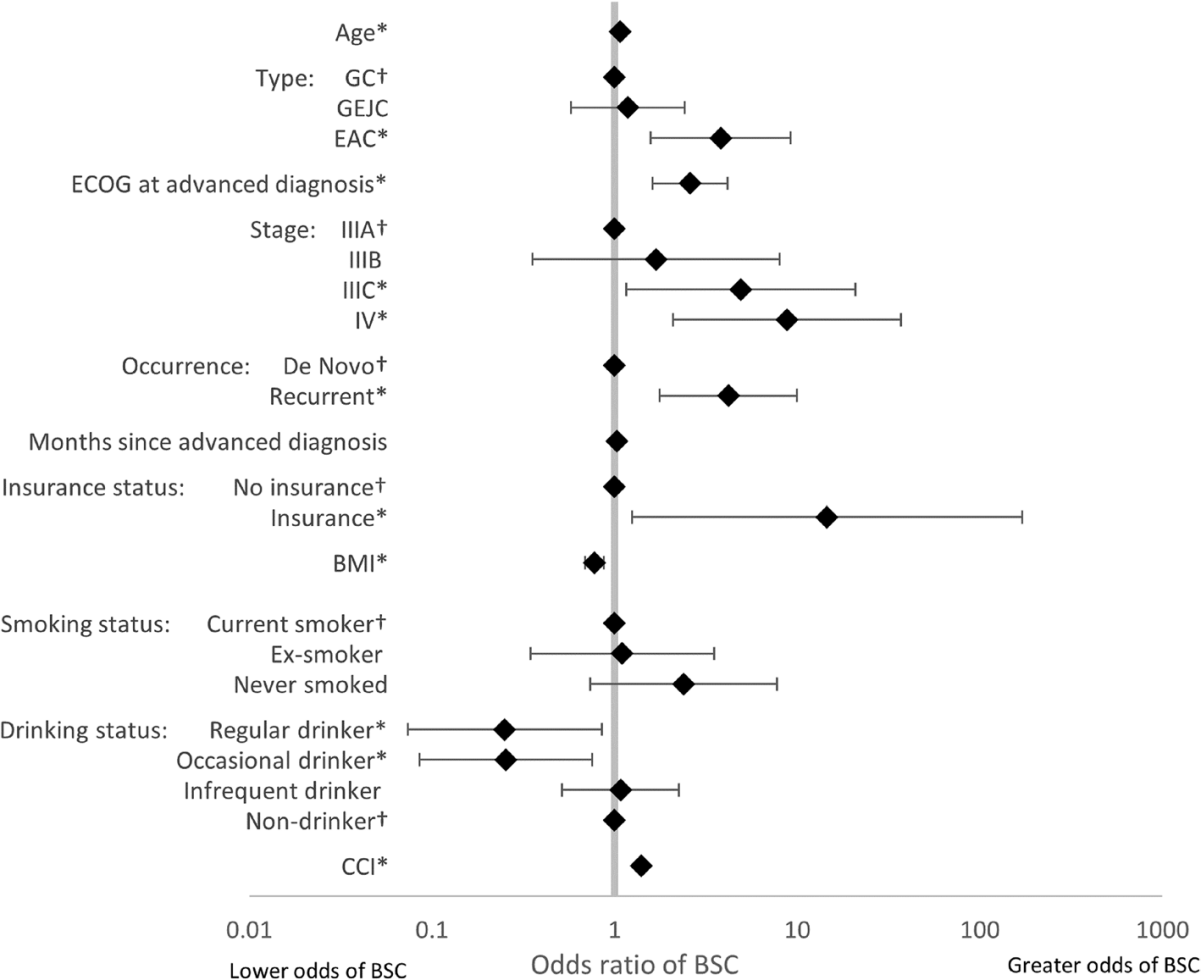
Drivers of active 1L vs BSC in patients with GC/GEJC/EAC, HER2 negative/unknown, on first-line treatment. 1L, first-line; BSC, best supportive care; BMI, Body Mass Index; CCI, Charlson Comorbidity Index; EAC, esophageal adenocarcinoma; ECOG, Eastern Cooperative Oncology Group; GC, gastric cancer; GEJC, gastroesophageal junction cancer. * *p* < 0.05, † base category. Regular heavy drinker and binge drinker omitted due to perfect prediction of no active 1L treatment

**Fig. 5:**
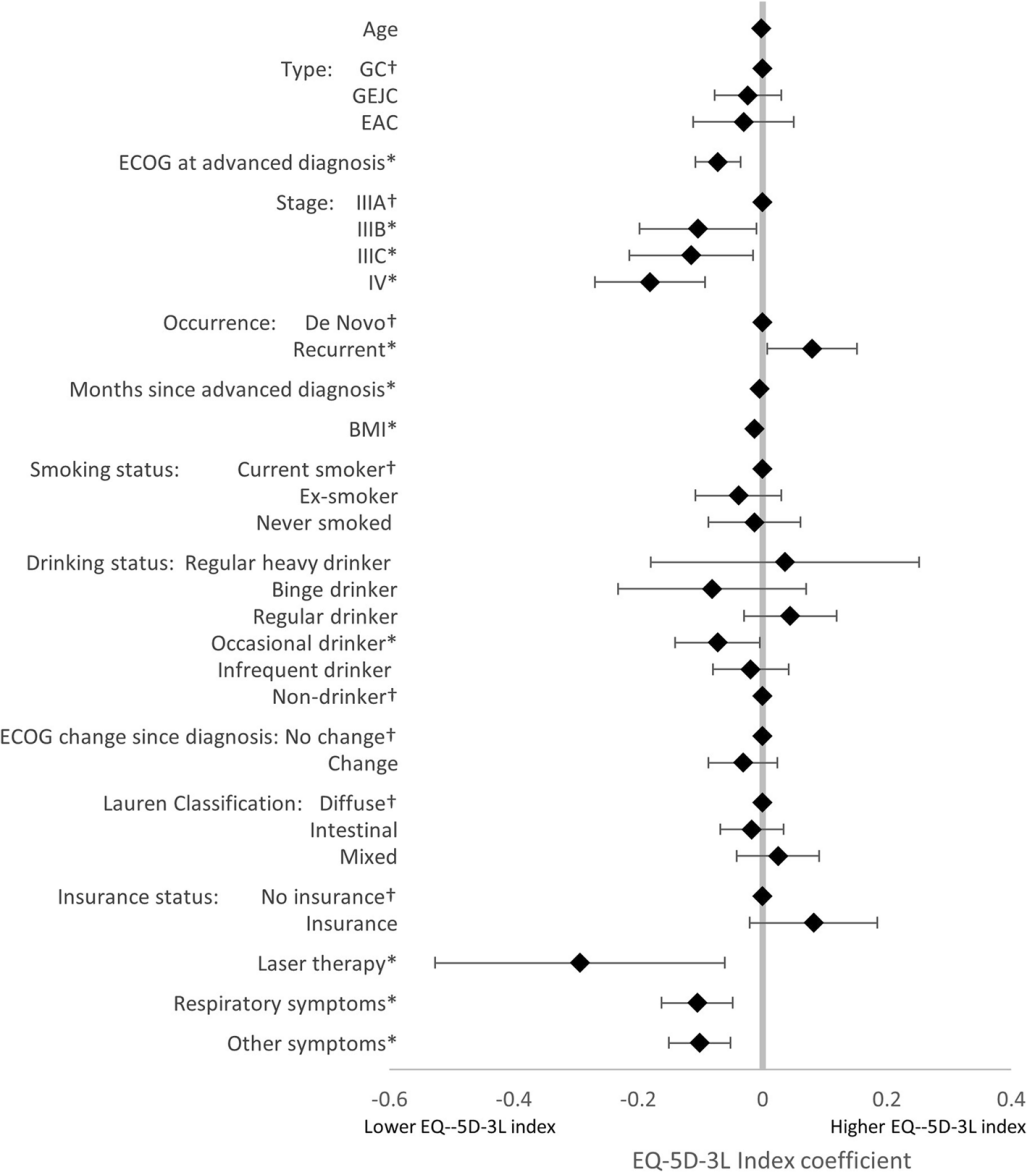
Drivers of EQ-5D-3L utility index scores in patients with GC/GEJC/EAC, HER2 negative/unknown, on first-line treatment. BMI, Body Mass Index; EAC, esophageal adenocarcinoma; ECOG, Eastern Cooperative Oncology Group; EQ-5D-3L, EuroQol 5-Dimension 3-Level Questionnaire; GC, gastric cancer; GEJC, gastroesophageal junction cancer. * *p* < 0.05, † base category. Respiratory symptoms include chest infection, shortness of breath, chest pain, persistent coughing, pleural effusion, sputum. Other symptoms include weakness and fatigue, jaundice, acute bleeding, headaches, anemia, other pain, other

**Fig. 6:**
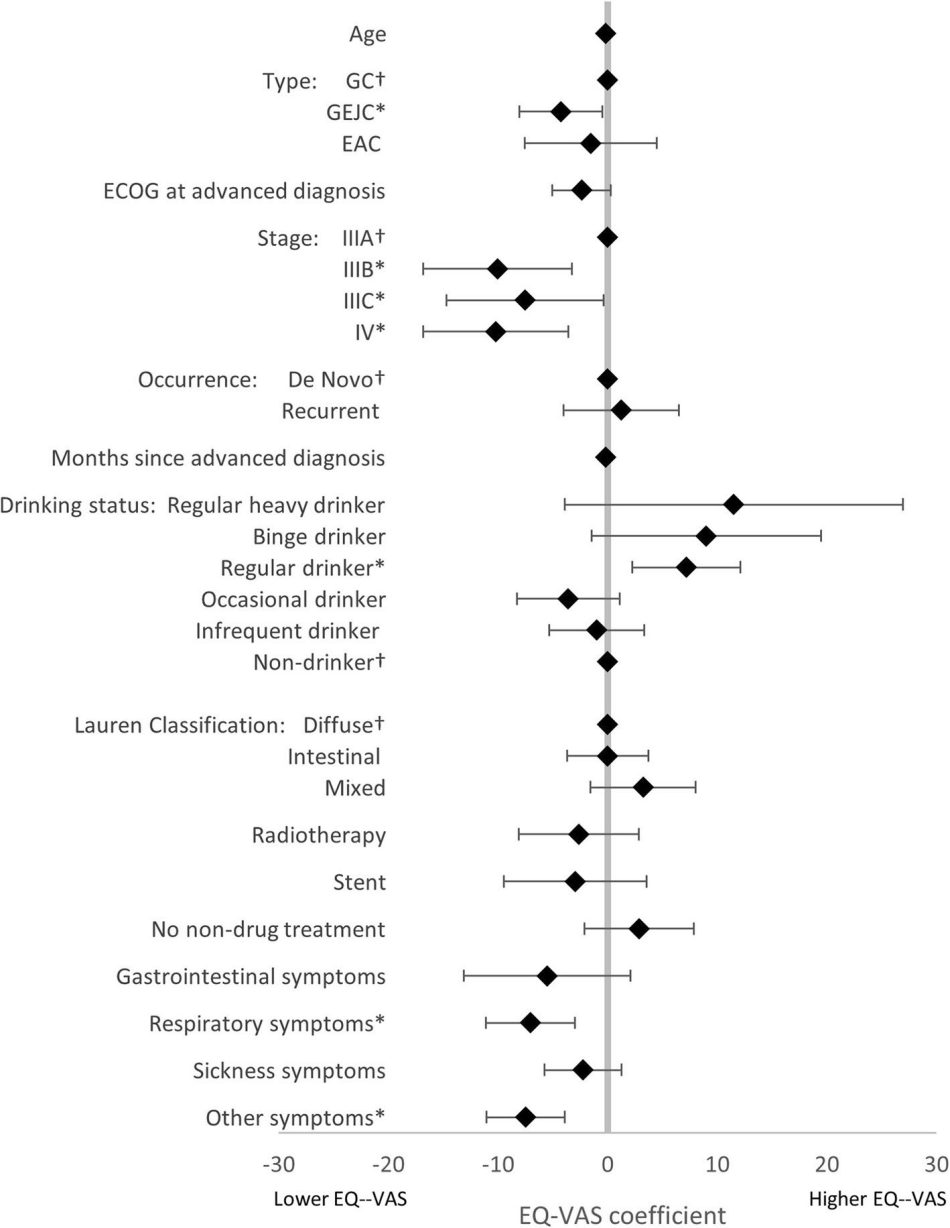
Drivers of EQ-VAS in patients with GC/GEJC/EAC, HER2 negative/unknown, on first-line treatment. EAC, esophageal adenocarcinoma; ECOG, Eastern Cooperative Oncology Group; EQ-VAS, EuroQol-Visual Analogue Scale; GC, gastric cancer; GEJC, gastroesophageal junction cancer. * *p* < 0.05, † base category. Gastrointestinal symptoms include indigestion, heartburn, reflux, bloating, flatulence, distended abdomen, early satiety, abdominal discomfort, loss of appetite, unexplained weight loss, melena, dysphagia, and odynophagia. Respiratory symptoms include chest infection, shortness of breath, chest pain, persistent coughing, pleural effusion, sputum. Sickness symptoms include nausea, vomiting, diarrhea. Other symptoms include weakness and fatigue, jaundice, acute bleeding, headaches, anemia, other pain, other

**Fig. 7:**
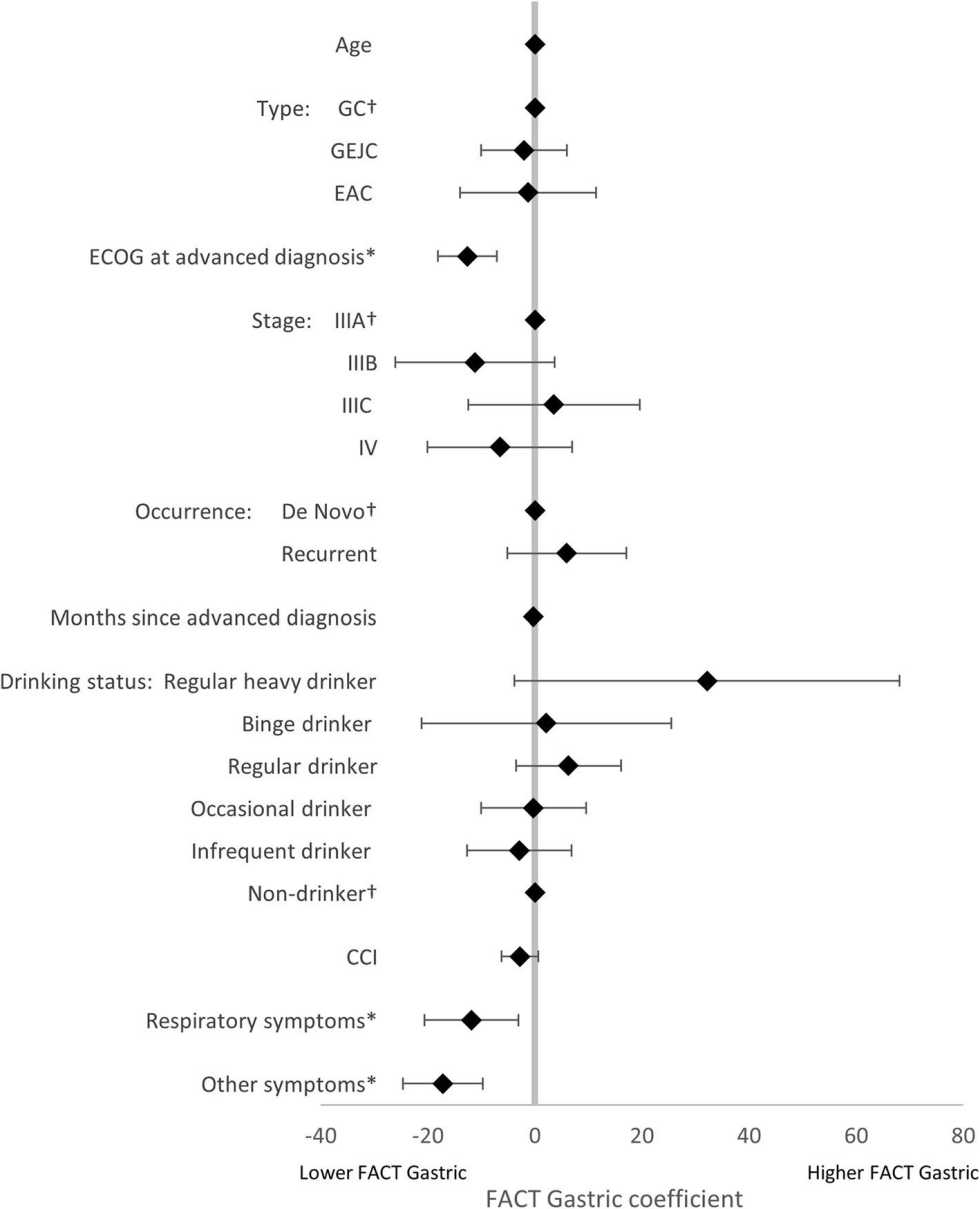
Drivers of FACT-Ga in patients with GC/GEJC/EAC, HER2 negative/unknown, on first-line treatment. CCI, Charlson Comorbidity Index; EAC, esophageal adenocarcinoma; ECOG, Eastern Cooperative Oncology Group; FACT-Ga, Functional Assessment of Cancer Therapy-Gastric Cancer; GC, gastric cancer; GEJC, gastroesophageal junction cancer. * *p* < 0.05, † base category. Respiratory symptoms include chest infection, shortness of breath, chest pain, persistent coughing, pleural effusion, sputum. Other symptoms include weakness and fatigue, jaundice, acute bleeding, headaches, anemia, other pain, other

**Fig. 8:**
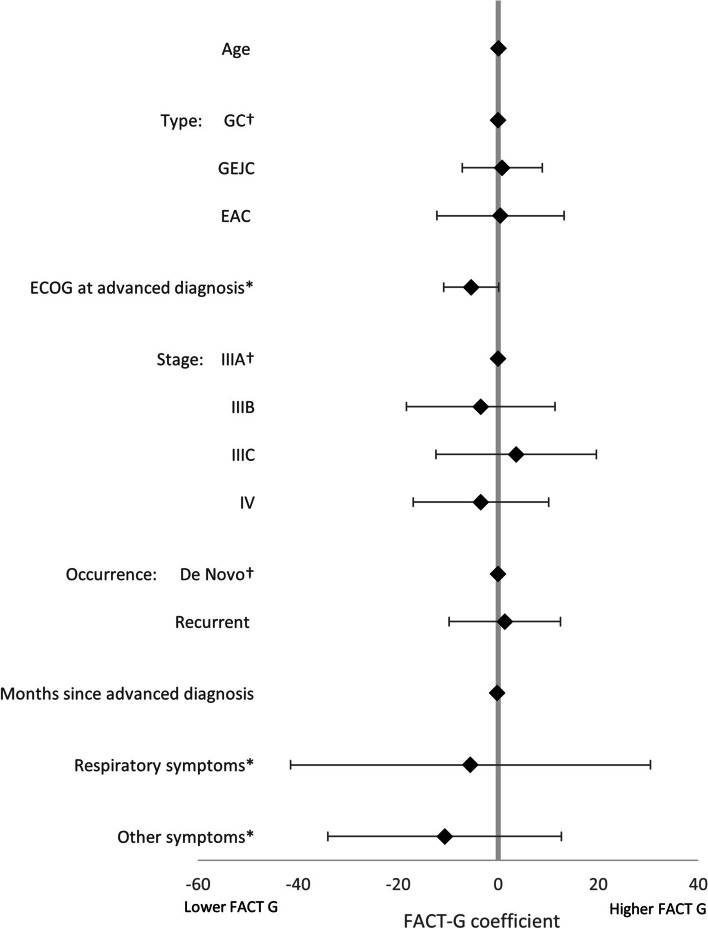
Drivers of FACT-G in patients with GC/GEJC/EAC, HER2 negative/unknown, on first-line treatment. EAC, esophageal adenocarcinoma; ECOG, Eastern Cooperative Oncology Group; FACT-G, Functional Assessment of Cancer Therapy-General; GC, gastric cancer; GEJC, gastroesophageal junction cancer. * *p* < 0.05, † base category. Respiratory symptoms include chest infection, shortness of breath, chest pain, persistent coughing, pleural effusion, sputum. Other symptoms include weakness and fatigue, jaundice, acute bleeding, headaches, anemia, other pain, other

LASSO regression analysis, used to identify the drivers of treatment (1L vs BSC), found that, of the non-forced predictors, BMI, smoking status, drinking status, and comorbidity burden were associated with treatment decisions. An increase in all predictors was associated with greater likelihood of receiving BSC, except for BMI and more frequent alcohol consumption (Fig. 4). LASSO regression analysis exploring the drivers of HRQoL in patients on active 1L therapy, together with the forced predictors, found that the following non-forced predictors were associated with HRQoL outcomes, and were therefore included in linear regression models: BMI (EQ-5D-3L utility index only), smoking status (EQ-5D-3L utility index only), drinking status (EQ-5D-3L utility index, EQ-VAS, and FACT-Ga), Lauren classification (EQ-5D-3L utility index and EQ-VAS), ECOG change (EQ-VAS only), radiotherapy (EQ-VAS only), comorbidity burden (FACT-Ga only), laser therapy (EQ-5D-3L utility index only), stent (EQ-VAS only), no nonpharmacological treatments (EQ-VAS only), gastric symptoms (EQ-VAS only), respiratory symptoms (all), sickness symptoms (EQ-VAS only), and other symptoms (all). Linear regression model coefficients are shown in Figs. 5, 6, 7 and 8. Further bivariate analyses exploring the effect of disease occurrence, de novo or recurrent, on each dimension of the EQ-5D-3L utility index showed that there were no significant differences associated except with usual activities (*p* < 0.001), where a lower proportion of patients with recurrent disease tended to report extreme problems performing their usual activities. Analysis on the impact of drinking alcohol on FACT G dimension scores showed no significant differences, however, heavy drinkers tended to have lower scores in functional well-being and gastric cancer subscales (*p* = 0.0529 and 0.0052, respectively).

## Discussion

In patients with advanced GC, although improved survival remains the main goal, PROs characterizing HRQoL are valuable in aiding treatment decisions. However, because only a few studies in patients with GC have assessed HRQoL data in conjunction with survival outcomes, the understanding and interpretation of these instruments remain limited [26].

In recent years, survival rates for patients with gastroesophageal adenocarcinomas have significantly improved in countries where screening programs have been implemented, suggesting that efforts to improve survival should focus on early diagnosis. The current guideline-recommended treatment of advanced HER2 negative gastroesophageal adenocarcinomas is 1L dual chemotherapy consisting of a fluoropyrimidine plus platinum, with dual therapy, for example, ramucirumab plus paclitaxel, as the 2L treatment [27–29]. In the third-line (3L) setting, nivolumab was shown to prolong overall survival in patients with advanced GEJC/GC after progression of at least two prior therapy regimes and has been approved for 3L treatment in Asia (ATTRACTION-2 trial) [30]. Since the collection of this data in 2019, 1L treatment comprised of a combination of nivolumab plus chemotherapy has recently been approved by the European Commission for use in patients with HER2 negative advanced or metastatic GC, GEJ, or EAC [31]. Therefore, HER2 testing has become even more important as personalized treatment options will now be available for all patients where HER2 status is known.

A patient’s chances of receiving BSC may differ depending on lifestyle and clinical factors. For example, we found that ECOG status, disease occurrence (recurrent cancer), smoking status (never smoked) and comorbidities were all predictors of BSC use in patients, while patients with higher BMIs and those that were regular/ heavy drinkers were less likely to receive BSC. Our findings are consistent with other literature that also cited poor ECOG status and comorbidities in colorectal and non-small cell lung cancer as being key drivers for patients receiving BSC alone [32, 33]. This is likely because certain comorbidities are exacerbated by anticancer drugs including pulmonary disease, psychiatric conditions and dementia [33], and those with a poor ECOG status (above 2) were found to be less likely to receive anticancer therapy due to the negative prognosis associated with having a poor ECOG status [34]. We also found that patients with insurance tended to be more likely to receive BSC. This was surprising given that BSC is a much more affordable option than other types of therapy [35], however, this might have been a result of the age group of the patients in our study, the majority of whom would be retired and therefore may be less likely to have comprehensive healthcare cover.

Improved survival among patients with metastatic GC with overexpression of HER2 has made anti-HER2 agents such as trastuzumab the standard of care for this population, and testing is generally recommended for all patients with GC/GEJC/EAC. However, because GC has a relatively low incidence in Western countries and as much as 75–85% were HER2 negative or unknown, HER2-testing, and the administration of anti-HER2 therapy to eligible patients might be suboptimal in some countries, leading to many of these patients not receiving the benefits of personalized treatment. In a recent study in the Netherlands, the proportion of patients tested was shown to increase over time, from 18% in 2010 to 88% in 2016, with a median overall survival increase in patients with HER2 positive, negative and unknown status of 9.8, 7.4, and 7.6 months, respectively [36]. These findings are further evidence that HER2 testing can allow patients’ treatment to be tailored to their specific status and improve the prognosis in a group of patients currently experiencing unmet need.

Whilst respondents in our study reported a successful response to 1L therapy, overall, patients had a worse health status than the general population as indicated by lower scores on the EQ-5D-3L utility index, the EQ-VAS and the FACT-G. Our results indicated that recurrent cancer had a less negative impact on EQ5D than de novo cases overall. However, this is likely explained by the fact that a lower proportion of respondents with recurrent cancer reported the extreme cases (for e.g., “I am confined to bed”, “I am unable to wash”, “I have extreme pain”) on most of the dimensions compared with de novo cases. While these differences were not statistically significant, in four out of five questions, when the individual effects of the dimensions were analyzed together, they may have led to the positive coefficient of recurrent cancer we found. In a healthcare system that includes medical environment, culture, sex, and comorbidities, even if the same treatment is available, the outcome in the individual patient is different [37]. In our study, the FACT-Ga, which combines the FACT-G with a 19-item gastric cancer subscale, showed significant impairment in HRQoL in patients with GC/GEJC/EAC compared to the general population. However, each country is unique, and although factors have been found to be associated with the HRQoL measured in this study when combining data from all regions, these drivers/factors may not apply in the individual countries.

The most frequently affected daily activities in patients with cancer are considered to be personal hygiene, housework, shopping, walking, and transportation [38] and, although the Katz Index indicated little impairment in our study population, patients nevertheless reported a high level of interference in their daily activities and their social lives due to their cancer.

There is an unmet need for the introduction of a novel therapy that is effective in the 1L treatment of GC/GEJC/EAC soon after diagnosis, particularly for patients whose HER2 status is negative or unknown, with a prospect of achieving improved quality, as well as quantity, of life in these patients. Making HER2 testing more available and more accessible across Europe and the US will enable patients to receive personalized and targeted treatment to increase overall survival in groups of patients with unmet need.

A number of strengths and limitations exist given the methodology. This was a non-interventional survey, with clinicians providing data for differing numbers of patients depending on the size of the advanced GC/GEJC/EAC consulting patient population. A limitation of this approach may be that the patient sample was not evenly distributed across the sites and might be weighted towards those sites with a large population of patients with advanced GC/GEJC/EAC patients. Furthermore, participants were encouraged, but not required, to complete all forms. As a result of the dependence on accurately completed questionnaires, the base sizes fluctuated across different variables. Finally, eligible patients were selected by physicians on a consecutive basis from the point of physician enrolment into the study. and it is therefore likely that patients who visited their physician more frequently were also more likely to have been included in the study. As the dataset is point-in-time, we were unable to make any conclusions about causal relationships, however, identification of significant associations was possible.

The strength of the survey is that it reflects real-world clinical practice and provides an insight into the daily management of advanced GC/GEJC/EAC and how this impacts patients’ well-being and HRQoL. Since this survey involved a relatively high number of clinicians from different countries, the sample is likely to be representative of the overall population of patients with advanced GC/GEJC/EAC in those countries*.*

## Conclusions

In patients with advanced GC/GEJC/EAC, for whom prognosis is poor, screening for HER2 status, as well as PROs, such as HRQoL, are valuable in aiding treatment decisions. The introduction of HER2 testing and appropriate therapy soon after diagnosis has the prospect of achieving improved quality, as well as quantity, of life in these patients.

## Data Availability

All data, i.e. methodology, materials, data and data analysis, that support the findings of this survey are the intellectual property of Adelphi Real World. All requests for access should be addressed directly to Jennifer Hall at Jennifer.Hall@adelphigroup.com. Jennifer Hall is an employee of Adelphi Real World.

## Abbreviations

1L: First-line
BMI: Body Mass Index
BSC: Best supportive care
CCI: Charlson Comorbidity Index
EAC: Esophageal adenocarcinoma
ECOG: Eastern Cooperative Oncology Group
eCRF: Electronic case report form
EphMRA: European Pharmaceutical Marketing Research Association
EQ-5D-3L: EuroQol 5-Dimension 3-Level Questionnaire
EQ-VAS: EuroQol-Visual Analogue Scale
ESCC: Esophageal squamous cell carcinoma
EWB: Emotional well-being
FACT-G: Functional Assessment of Cancer Therapy-General
FACT-Ga: Functional Assessment of Cancer Therapy-Gastric Cancer
FWB: Functional well-being
GC: Gastric cancer
GEJC: Gastroesophageal junction cancer
HER2: Human epidermal growth factor receptor 2
IQR: Interquartile range
PRO: Patient-reported outcome
PWB: Physical well-being
HRQoL: Quality of life
SD: Standard deviation
SWB: Social/family well-being
TNM: Tumor, Nodes, Metastases
UK: United Kingdom
US: United States [of America]
